# Supplementary material for: NMPylation and de-NMPylation of SARS-CoV-2 nsp9 by the NiRAN domain
Source: Nucleic Acids Res. 2021 Aug 5;49(15):8822–35. doi: 10.1093/nar/gkab677 (PMC8385902; doi:10.1093/nar/gkab677)
Supplement: gkab677_Supplemental_File [file gkab677_supplemental_file.pdf]

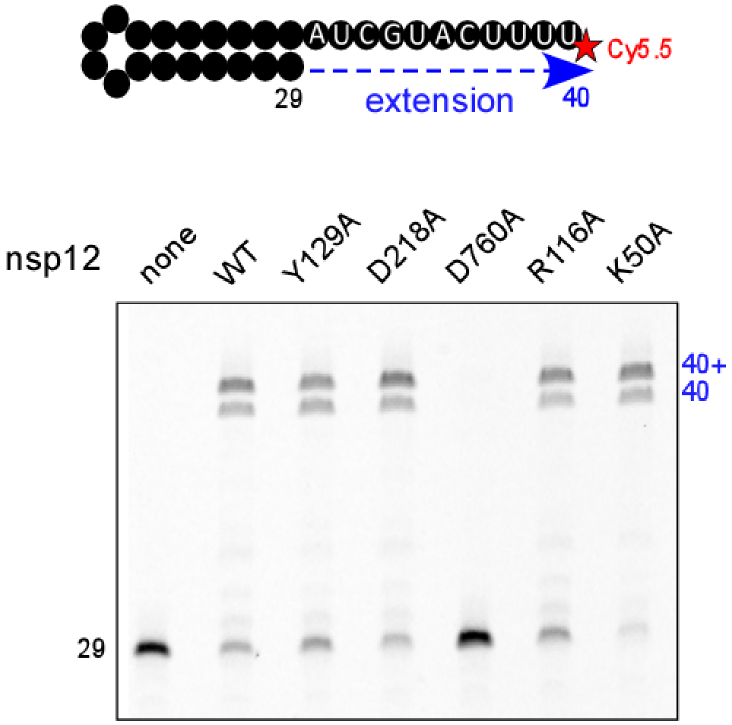

Supplementary Figure S1. RNA synthesis activities of nsp12 variants. RdRp holoenzymes nsp7•8_2•12 (WT or indicated mutants) were assembled and tested as described previously (1). A 29-nt RNA hairpin scaffold that contains Cy5.5 at the 5' end is extended by RdRp to produce a 40-nt product; additional extension is thought to be mediated by nsp8 after the completion of RNA synthesis (2). We use this assay, in parallel with the assay of NMP transfer by the NiRAN domain to assess the overall state of a mutant protein – we expect that a grossly misfolded Nsp12 would be defective in both activities.

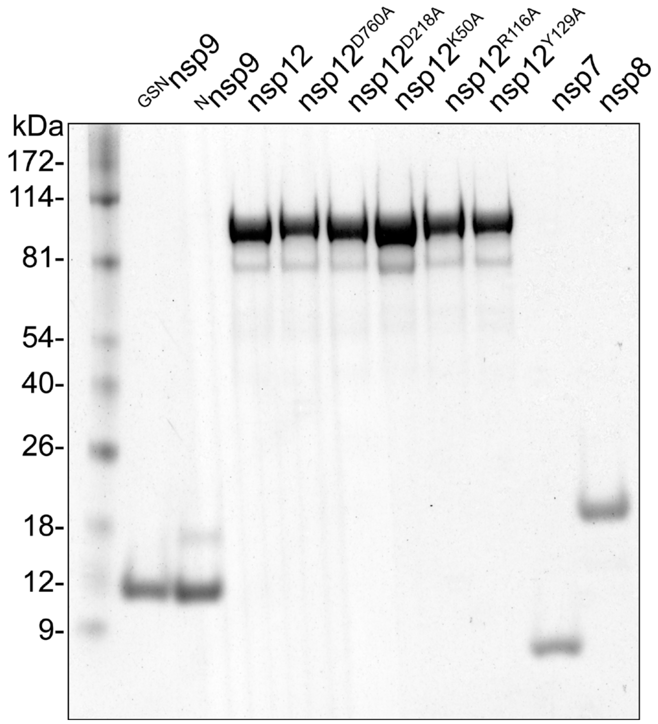

Supplementary Figure S2. SDS-PAGE of the purified nsp9 variants, nsp12 variants, nsp7, and nsp8.

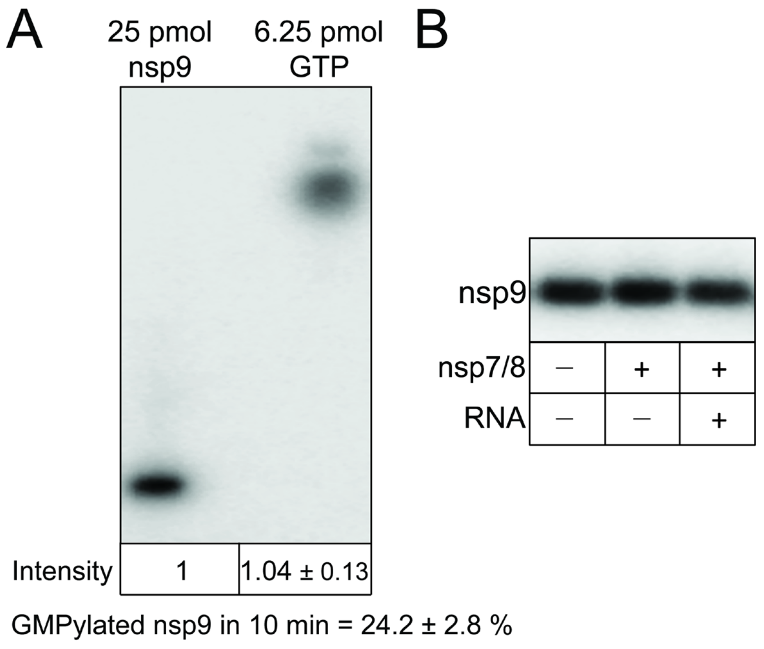

Supplementary Figure S3. The nsp12-mediated NMPylation of nsp9 is observed in the holoenzyme and the transcription complex. (A) GMPylation efficiency measurements. GMPylation reaction was performed as described in Materials and Methods for 10 minutes. 25 pmoles of GMPylated nsp9 were loaded on a denaturing gel and subjected to electrophoresis at 180 V for 25 min. Then 6.25 pmoles GTP (cold + radiolabeled) were loaded onto the gel, followed by further 5 min electrophoresis. Triplicate experiment was performed. (B) The NMPylation reaction was carried out by nsp12 alone as in **Fig. 1** (first lane), in the presence of nsp7 and nsp8 added at 1.5 and 3x molar excess relative to Nsp12, respectively, to assemble the RdRp holoenzyme nsp7•8_2•12 (second lane), and in a transcription complex assembled on the RNA scaffold shown in **Fig. S1** (third lane).

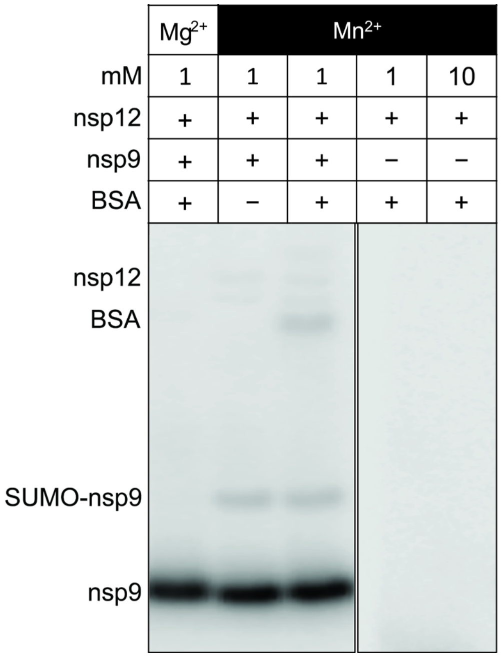

Supplementary Figure S4. nsp12 mediates fortuitous NMPylation in the presence of the Mn^{2+} ion. We have not observed nucleotidyl transfer by nsp12 to proteins other than nsp9 in the presence of either 1 or 2 mM Mg^{2+}thought to represent physiological cellular levels (3). However, we observed very inefficient NMPylation of nsp12 (**Fig. 1C**) and BSA at 1 mM Mn^{2+}. 5 µM BSA, 0.5 µM nsp12, and 5 µM nsp9 were incubated for 10 min in the NMPylation buffer (see Methods). An additional species visible on a gel is uncleaved SUMO-Nsp9 fusion (identified by MS analysis).

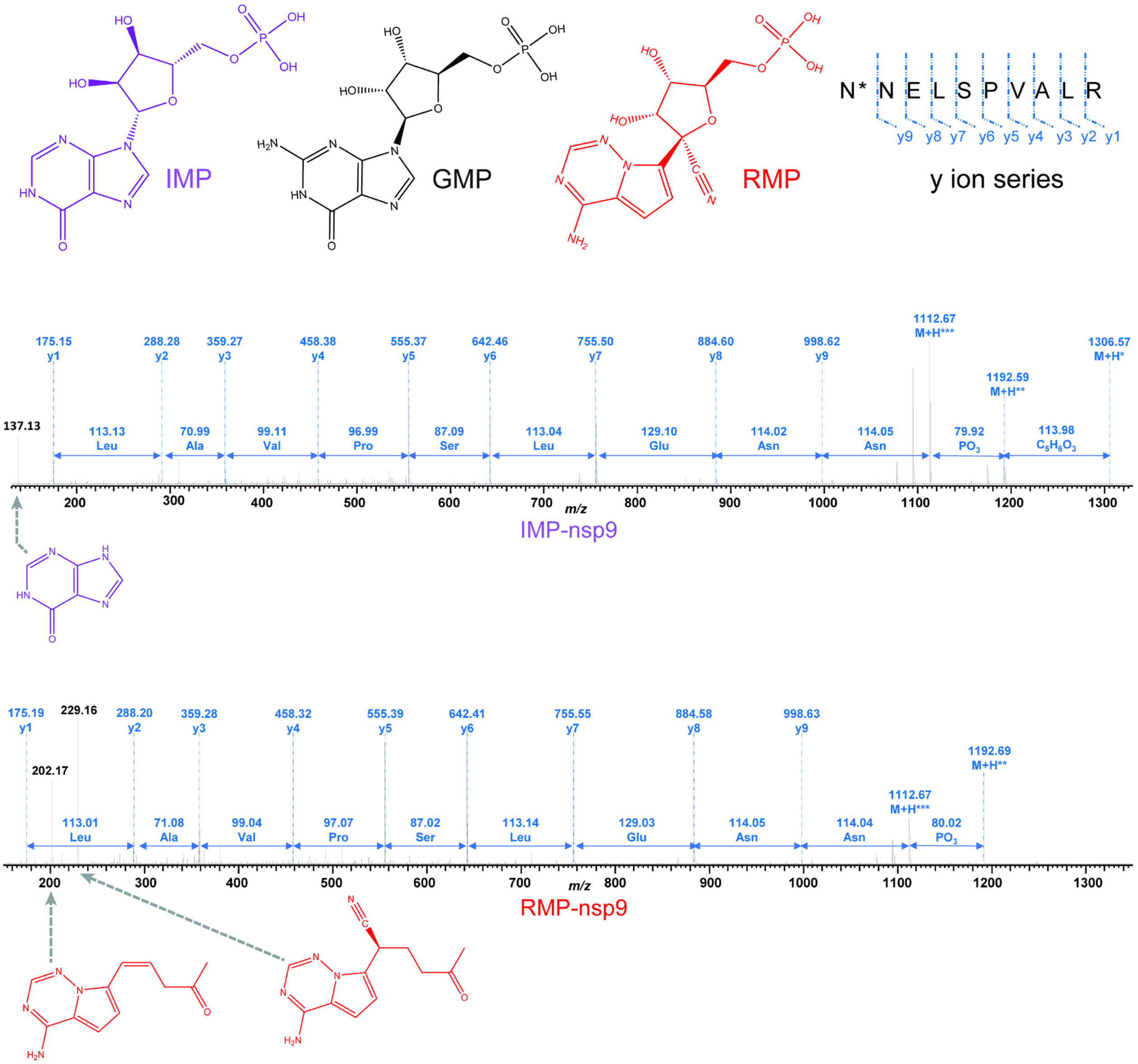

Supplementary Figure S5. Detection of modified nsp9 using LC–MS/MS. (Top) Structures of GMP analogs and y-ion series fragmentation for the N-terminal tryptic peptide of nsp9, with the asterisk indicating the known (4,5) GMP modification site. (Bottom) HCD peptide fragmentation for the same peptide modified with IMP or RMP at the indicated mass-to-charge ratio (m/z). Products appearing in addition the nsp9-derived products (slate) are indicated in black [IMP: 137.13; RMP: 202.17 and 229.16] and their corresponding chemical structures are shown below each fragmentation pattern. NMPylation reactions were caried out as described in methods, separated on denaturing protein gels, and stained. Protein bands corresponding to nsp9 were excised and analyzed at the Mass Spectrometry and Proteomics Facility, Comprehensive Cancer Center, the Ohio State University, as follows. The gel slices were washed with acetonitrile for 5 min, dried, and incubated sequentially in 50 mM NH_4HCO_3 with 5 mg/ml DTT and 15 mg/ml iodoacetamide The gel slices were alternately incubated with 50 mM

NH_4HCO_3 and acetonitrile, twice, for 5 min each and completely dried in vacuo. Dried gel slices were rehydrated with 25 μL of 50 mM NH_4HCO_3 and digested with sequencing grade-modified trypsin (Promega, Madison WI) overnight. The peptides were extracted with 50 % acetonitrile and 5 % formic acid three times, followed by a final extraction with acetonitrile. The extracted pool was completely dried in a vacuo and peptides were resuspended in 20 μL of 50 mM acetic acid. **Orbitrap Fusion:** Capillary-liquid chromatography-nanospray tandem mass spectrometry was performed on a Thermo Scientific orbitrap Fusion mass spectrometer equipped with an nanospray FAIMS Pro™ Sources operated in positive ion mode. Samples (6.4 µL) were separated on an easy spray nano column (Pepmap^{TM} RSLC, C18 3µ 100A, 75µm X250mm Thermo Scientific) using a 2D RSLC HPLC system from Thermo Scientific. Each sample was injected into the µ-Precolumn Cartridge (Thermo Scientific) and desalted with 0.1 % formic acid in water for 5 min. The injector port was then switched to inject and the peptides were eluted off of the trap onto the column. Mobile phase A was 0.1 % Formic Acid in water; mobile phase B was acetonitrile with 0.1 % formic acid; flow rate was set at 300 nL/min. Mobile phase B was increased from 2 to 16 % in 45 min, to 25 % in 10 min, to 90 % in 1 min and then kept at 90 % for 2 min before being brought back quickly to 2 % in 1 min. The column was equilibrated at 2 % B for 15 min before the next sample injection. MS/MS data were acquired with a spray voltage of 1.95 KV and a capillary temperature of 305 °C is used. The scan sequence of the mass spectrometer was based on the preview mode data dependent TopSpeed™ method: the analysis was programmed for a full scan recorded between *m/z* 375-1500 and a MS/MS scan to generate product ion spectra to determine amino acid sequence in consecutive scans starting from the most abundant peaks in the spectrum in the next 3 seconds. To achieve high mass accuracy MS determination, the full scan was performed at FT mode and the resolution was set at 120,000 with internal mass calibration. Three compensation voltages (cv=-50, -65 and -80) were used for sample acquisition. The AGC Target ion number for FT full scan was set at 4×10^5 ions, maximum ion injection time was set at 50 ms, and micro scan number was set at 1. MSn was performed using HCD in ion trap mode to ensure the highest signal intensity of MSn spectra. The HCD collision energy was set at 32 %. The AGC Target ion number for ion trap MSn scan was set at 3.0E4 ions, maximum ion injection time was set at 35 ms, and micro scan number was set at 1. Dynamic exclusion is enabled with a repeat count of 1 within 60 s and a low mass width and high mass width of 10 ppm. Data were searched using Mascot Daemon by Matrix Science version 2.7.0 (Boston, MA) via ProteomeDiscoverer (version 2.4 Thermo Scientific,) and the database searched against the most recent Uniprot databases. The mass accuracy of the precursor ions was set to 10 ppm, accidental pick of 1 ^{13}C peaks was also included into the search. The fragment mass tolerance was set to 0.5 Da. Carbamidomethylation (Cys) is used as a fixed modification and considered variable modifications were oxidation (Met) and deamidation (N and Q). Four missed cleavages for the enzyme were permitted. A decoy database was also searched to determine the false discovery rate (FDR) and peptides were filtered according at 1 % FDR. Any modified peptides are manually checked for validation.

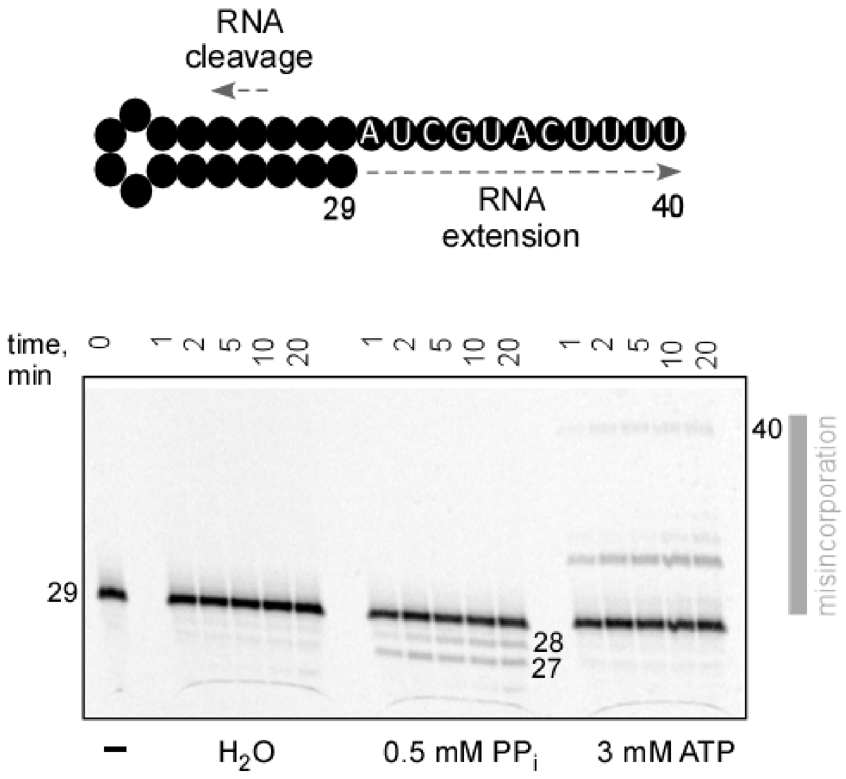

Supplementary Figure S6. Non-cognate substrate does not induce RNA cleavage by SARS-CoV-2 RdRp. The reactions were carried out in the presence of 5 mM Mg^{2+} to avoid titrating the metal ion by ATP. We observe synthesis of full-length 40-mer product, indicating that RdRp misincorporates AMP in place of UMP (1st position), GMP (3rd and 7th positions) and CMP (4th position) at high ATP concentrations.

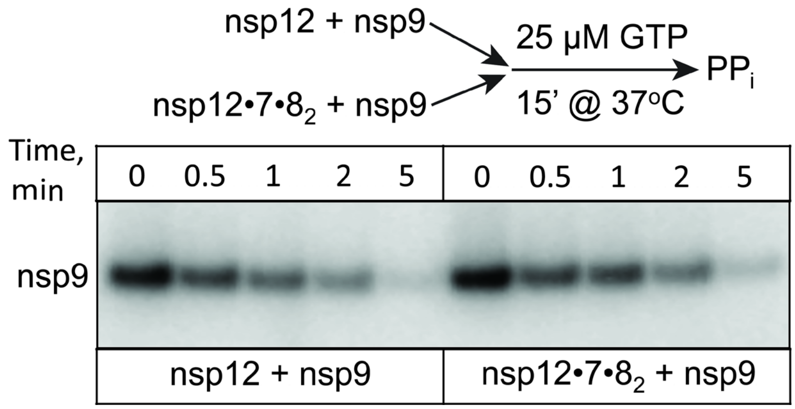

Supplementary Figure S7. PP_i-induced de-GMPylation of nsp9 by nsp12 and nsp7•8_2•12 holoenzyme.

The reactions were carried out in parallel as described in the Methods section.

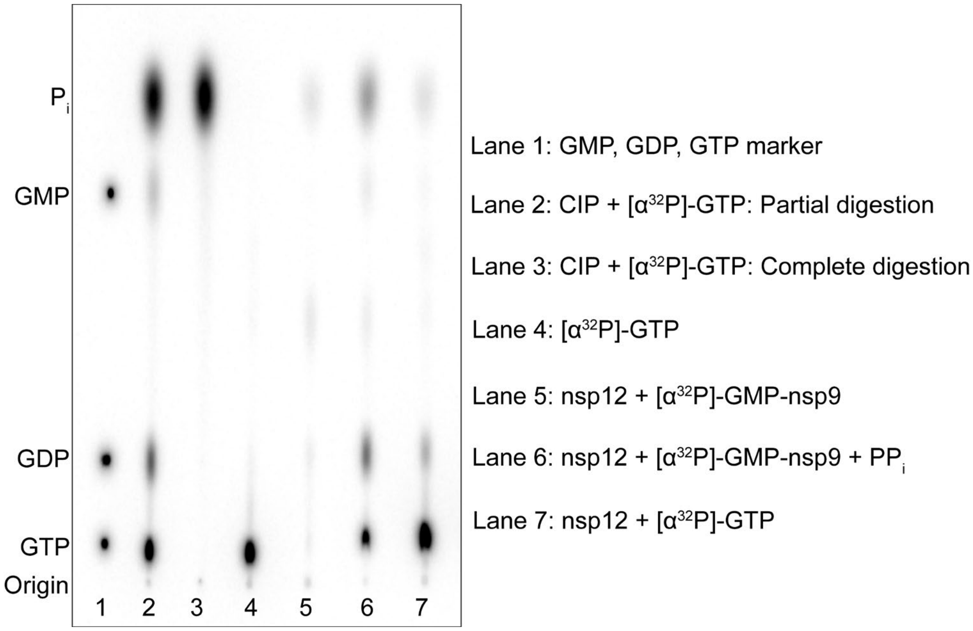

Supplementary Figure S8. PP_i mediated reversal reaction of nsp9 GMPylation. Lanes 1 – 4 are used as markers. Lane 1: Cold GMP, GDP, and GTP were visualized by fluorescence at 254 nm and the corresponding positions were marked with a [α^{32}P]-GTP solution. Lanes 2 and 3: Partially and completely digested by Quick CIP (calf intestinal alkaline phosphatase, NEB, Cat#M0525S) [α^{32}P]-GTP. Lane 4: [α^{32}P]-GTP. To observe the reversal reaction, 2 µM nsp12, 20 µM nsp9, 25 µM GTP, and 30 µCi [α^{32}P]-GTP were incubated in NMPylation buffer (25 mM HEPES, pH 7.5, 15 mM KCl, 5 % glycerol, 2 mM $MgCl_2$, 2 mM DTT) for 30 min, followed by passing through Illustra MicroSpin G-50 Columns (GE Healthcare) to remove unincorporated [α^{32}P]-GTP. Then H_2O (lane 5) or 0.5 mM PP_i (lane 6) was added and the reactions were incubated for 15 min at 37 ^oC. Reactions were stopped by adding one volume of phenol:chloroform:isoamyl alcohol (25:24:1, v/v). Samples were analyzed by thin layer chromatography on PEI-cellulose plates (Millipore Sigma, Cat#1.05579) in 1 M NaCl. Following the addition of PP_i, we observed the formation of GTP, which is consistent with the direct reversal of NMPylation, but also GDP and P_i. Incubation of 0.5 µM nsp12 with 10 µCi [α^{32}P]-GTP for 15 min at 37 °C (lane 7) also resulted in production of GDP and P_i (lane 7), suggesting that nsp12 has a somewhat unusual intrinsic hydrolytic activity; we intend to investigate this mechanism in the future.

Supplementary Table S1. Plasmids

Name	Key features/sequence	Source; Addgene#
pIA1362	T7 promoter–His_8-GB1-TEV-nsp7	(1); 166860
pIA1363	T7 promoter–His_8-GB1-TEV-nsp8	(1); 166861
pIA1364	T7 promoter–His_8-GB1-TEV-nsp9: expresses ^{GSN}nsp9	This work; 172401
pIA1400	T7 promoter–His_{10}-SUMO-nsp12	This work; 172519
pIA1402	T7 promoter–His_{10}-SUMO-nsp12[Y129A]	(1); 172403
pIA1405	T7 promoter–His_{10}-SUMO-nsp12[D218A]	(1); 172404
pIA1408	T7 promoter–His_{10}-SUMO-nsp12[D760A]	This work; 172405
pIA1414	T7 promoter–His_{10}-SUMO-nsp9: expresses the native ^{N}Nsp9	This work; 172402
pIA1420	T7 promoter–His_{10}-SUMO-nsp12[R116A]	This work; 172406
pIA1421	T7 promoter–His_{10}-SUMO-nsp12[K50A]	This work; 172407

Supplementary references

1. Wang, B., Svetlov, V., Wolf, Y.I., Koonin, E.V., Nudler, E. and Artsimovitch, I. (2021) Allosteric activation of SARS-CoV-2 RdRp by remdesivir triphosphate and other phosphorylated nucleotides. *mBio*, **12**, e0142321.
2. Tvarogová, J., Madhugiri, R., Bylapudi, G., Ferguson, L.J., Karl, N. and Ziebuhr, J. (2019) Identification and Characterization of a Human Coronavirus 229E Nonstructural Protein 8-Associated RNA 3'-Terminal Adenylyltransferase Activity. *J Virol*, **93**, e00291-00219.
3. Gottesman, M.E., Chudaev, M. and Mustaev, A. (2020) Key features of magnesium that underpin its role as the major ion for electrophilic biocatalysis. *FEBS J*, **287**, 5439-5463.
4. Conti, B.J., Leicht, A.S., Kirchdoerfer, R.N. and Sussman, M.R. (2021) Mass spectrometric based detection of protein nucleotidylation in the RNA polymerase of SARS-CoV-2. *Commun Chem*, **4**.
5. Slanina, H., Madhugiri, R., Bylapudi, G., Schultheiss, K., Karl, N., Gulyaeva, A., Gorbalenya, A.E., Linne, U. and Ziebuhr, J. (2021) Coronavirus replication-transcription complex: Vital and selective NMPylation of a conserved site in nsp9 by the NiRAN-RdRp subunit. *Proc Natl Acad Sci U S A*, **118**.
